# Bed net use among school-aged children after a universal bed net campaign in Malawi

**DOI:** 10.1186/s12936-016-1178-9

**Published:** 2016-02-29

**Authors:** Andrea G. Buchwald, Jenny A. Walldorf, Lauren M. Cohee, Jenna E. Coalson, Nelson Chimbiya, Andy Bauleni, Kondwani Nkanaunena, Andrew Ngwira, Atupele Kapito-Tembo, Don P. Mathanga, Terrie E. Taylor, Miriam K. Laufer

**Affiliations:** Institute for Global Health, University of Maryland School of Medicine, 685 W. Baltimore St. HSF-1 Room 480, Baltimore, MD 21201 USA; Department of Epidemiology, University of Michigan School of Public Health, Ann Arbor, USA; Malaria Alert Center, University of Malawi College of Medicine, Blantyre, Malawi; College of Osteopathic Medicine, Michigan State University, East Lansing, MI USA

**Keywords:** ITNs, Net distribution, School-aged children, Malawi

## Abstract

**Background:**

Recent data from Malawi suggest that school-aged children (SAC), aged 5–15 years, have the highest prevalence of *Plasmodium falciparum* infection among all age groups. They are the least likely group to utilize insecticide-treated nets (ITNs), the most commonly available intervention to prevent malaria in Africa. This study examined the effects of a universal ITN distribution campaign, and their durability over time in SAC in Malawi. This study identified factors that influence net usage among SAC and how these factors changed over time.

**Methods:**

Cross-sectional surveys using cluster random sampling were conducted at the end of each rainy and dry season in southern Malawi from 2012 to 2014; six surveys were done in total. Mass net distribution occurred between the first and second surveys. Data were collected on household and individual net usage as well as demographic information. Statistical analyses used generalized linear mixed models to account for clustering at the household and neighbourhood level.

**Results:**

There were 7347 observations from SAC and 14,785 from young children and adults. SAC used nets significantly less frequently than the rest of the population (odds ratio (OR) from 0.14 to 0.38). The most important predictors of net usage among SAC were a lower ratio of people to nets in a household and higher proportion of nets that were hanging at the time of survey. Older SAC (11–15 years) were significantly less likely to use nets than younger SAC (5–10 years) [OR = 0.24 (95 % CI: 0.21, 0.28)]. The universal bed net campaign led to a statistically significant population-wide increase in net use, however net use returned to near baseline within 3 years.

**Conclusions:**

This study suggests that a single universal net distribution campaign, in combination with routine distribution through health clinics is not sufficient to cause a sustained increase in net usage among SAC. Novel approaches to ITN distribution, such as school-based distribution, may be needed to address the high prevalence of infection in SAC.

## Background

Efforts in sub-Saharan Africa to control and ultimately eliminate malaria focus on a repertoire of limited but proven interventions: indoor residual spraying (IRS), insecticide-treated bed nets (ITNs), and improved access to prompt malaria diagnosis and treatment with artemisinin-based combination therapy. Large-scale distribution of ITNs has been associated with reductions in parasite transmission and prevalence in some sub-Saharan African settings [1–5]. Like most malaria-endemic countries, Malawi has scaled up ITN distribution, and since 2005, long-lasting ITNs have been distributed without cost to children born in government health facilities, children receiving their first immunizations, and to pregnant women attending their first antenatal care visit in a government facility [6]. To improve access to bed nets, the Ministry of Health and its partners conducted the first nationwide universal ITN distribution campaign during 2012 with the goal of providing nets at a ratio of one net for every two people in the population at large.

Despite successful scale-up of interventions, the malaria burden in Malawi has not declined [7, 8]. Understanding the factors contributing to this apparent lack of effect is vital to determining the appropriate measures to bring about reduction of the malaria burden in Malawi. Current malaria control measures in Malawi, including ITN distribution, may have failed to produce the desired decrease in malaria transmission for a variety of reasons. Only 59.5 % of households reported owning at least one bed net in 2012, thus despite distribution through health clinics, lack of access to ITNs may be a large factor in high parasite prevalence [8]. The decay of the effectiveness of bed nets after a distribution campaign has also been linked both to decreased use and diminished efficacy at preventing transmission [9–12].

One possible explanation for the persistence of malaria infection is that ITNs may not be used by populations that are serving as reservoirs for infection and transmission. Walldorf et al. recently demonstrated that school-aged children aged 5–15 years old (SAC) have the highest prevalence of malaria infection compared to other age groups and are also the group least likely to sleep under a bed net [13].

This study sought to examine the factors associated with bed net use in this high prevalence population in Malawi. Cross-sectional surveys using cluster random sampling were conducted at the end of each rainy and dry season in southern Malawi from 2012 to 2014. A universal net distribution campaign occurred in Malawi in 2012 after the first survey. The goal of the campaign was to distribute nets to all households at a ratio of one net for every two people in the household. Data from these surveys was used to identify the correlates of net use in SAC, the age-specific impact of the universal bed net campaign on bed net use and the change in patterns of use after the distribution.

## Methods

### Survey

Cross-sectional household surveys were conducted in southern Malawi in Blantyre, Chikhwawa, and Thyolo districts at the end of the dry, low transmission season (September–October), as well as at the end of the rainy, high transmission season (April–May) from 2012 to 2014. The study design was described previously [13]. Districts were chosen to represent three distinct ecological settings in Malawi, an urban setting, a high-altitude region and a low altitude region. Ten enumeration areas (EAs), each containing one compact segment of 30 households, were selected in each of three districts [14]. The same selected households in each district were surveyed each season, and all households within a given EA were visited on a single day. Households were excluded if there were no adults over 18 years to provide consent or if the house had been abandoned or destroyed. If excluded, a household was replaced with another household adjacent to the compact segment where possible.

### Ethical treatment of human subjects

Prior to study initiation, permission to survey each village was provided by the village leaders. Written informed consent was obtained for all adults and children and assent was obtained for children age 13–17 years. All questionnaires were administered in the local language. The study received ethical approval from both the University of Maryland Baltimore and Michigan State University Institutional Review Boards and the University of Malawi College of Medicine Research and Ethics Committee.

### Study participants

Members of a household were defined as individuals who slept in the house for at least 2 weeks of the previous month. Interview data about all household members were collected from any consenting adult household member present, while specimens were collected from only those present at the time of survey. Although households in the same geographic area were selected in each survey, the specific sampled individuals varied from survey to survey depending on those present on the day of the survey. Sampling of the same individuals may have occurred across surveys. However, individual identifiers were not collected.

### Data collection and variable definitions

Questionnaires were adapted from the standardized Malaria Indicator Survey (MIS) tools. Data were collected on android-based tablets using OpenDataKit [15] and managed using Research Electronic Data Capture (REDCap) tools [16]. Data collected included household characteristics and socio-economic indicators, individual demographics, household net ownership and individual use, and information on recent IRS. Age categories were classified into three groups: young SAC (5–10 years old), old SAC (11–15 years old), and non-SAC (children under age five and adults over 15).

An individual was categorized as using a bed net if they were identified in the question “Which members of the household slept under this bed net last night?” A household wealth index was developed using standard indicators, including household construction materials and bicycle or cellular phone ownership; households in the study population were then broken into tertiles based on the wealth index score. Head of household education level was classified into four categories: never having attended school, attended (but did not complete) primary school, completed primary school, and some secondary education or greater. Average age of nets was calculated based on self-reported net age for each net in a household.

Transmission setting was classified into three categories: urban (Blantyre), rural moderate transmission settings and rural high transmission settings. Transmission setting was determined from EA malaria parasite prevalence in the total population during the survey. Malaria parasite prevalence was calculated from the average per cent of specimens positive by qPCR for malaria parasites in a given EA over the course of the six surveys. Moderate transmission EAs were defined as having less than 16 % parasitaemia prevalence. High transmission EAs had parasitaemia prevalence of 16 % or greater. These definitions were chosen from the median of EA prevalence from all surveys. A variable for EA infection prevalence at the time of survey was included in statistical models to adjust for local prevalence differences within transmission settings. Household net use was defined as the proportion of the household members reported to have slept under a net the night before the survey. Community net use was defined as the proportion of all individuals surveyed in the EA reported to have slept under a net the night before the survey.

### Statistical analysis

#### Predictors of net use

All analyses of predictors of SAC net use were restricted to the population of SAC from households that reported owning one or more net. Variables associated with SAC net use were determined using Chi square tests for individual surveys. Cochran–Armitage tests for trend were conducted to identify any trends in net use over time and Mantel–Haenszel Chi square tests were done to assess for population-wide associations stratified by survey. Variables tested included age, sex, household wealth tertile, head of household education level, transmission setting, presence of a child under 5 years of age in the household, proportion of nets to sleeping spaces, ratio of number of individuals to the number of nets in the household, proportion of nets observed hanging among all nets in the house at the time of survey, average age of nets in the house, and source of nets in the household. A stratified analysis was done by transmission setting since malaria transmission setting was assumed to be a modifier of malaria preventive behaviour.

Generalized linear mixed models (glimmix procedure) with a random intercept to account for hierarchical clustering by EA and household were used to determine significant predictors of SAC net use. All variables that were statistically significant at the alpha = 0.05 level from bivariate analysis were initially included in regression models. In multivariate regression, any variables that were not significant at the 0.05 level and did not significantly improve the model’s fit were removed. Individual models were constructed for each survey to identify any change in predictors over time. A final model, stratified by transmission setting, included survey as a categorical variable and any variables significant at three or more time points.

#### Association between age and net use over time

The association between age and net use in the population overall was assessed using Chi square tests for individual surveys. A difference in associations by survey was tested using Breslow-Day. A crude estimate of the prevalence odds ratio (OR) for net use comparing SAC to all other ages stratified by survey was obtained using the Mantel–Haenszel prevalence OR (MH-POR). A model for the adjusted OR of net usage comparing SAC to all other ages was constructed using significant predictors of net usage identified in the predictive model described previously.

Household net ownership and net use were compared between surveys using Chi square tests and a Cochran–Armitage test for trend. A between-season change in OR for net usage comparing SAC to all other ages was assessed by including an interaction term for survey*age into generalized linear mixed models. Interaction terms were considered significant if they had a *p* value of 0.05 or less. Statistical analysis was conducted in SAS 9.3 (Cary, NC, USA).

## Results

There were 22,132 observations included in the analysis, including 7347 observations among SAC. The population distribution between surveys did not vary significantly by transmission setting, age, head of household, education level, household size, or sex (Table 1).

**Table 1:** Population characteristics from six cross-sectional surveys (N = 22, 132)

Population characteristics	Rainy 2012	Dry 2012	Rainy 2013	Dry 2013	Rainy 2014	Dry 2014
N	3475	3873	3767	3680	3590	3747
Urban	1215	1320	1281	1259	1272	1331
Rural moderate transmission	1087	1237	1199	1155	1094	1142
Rural high transmission	1173	1316	1287	1266	1224	1274
Age category						
Less than 5 years (%)	17.7	17.7	17.8	16.8	17.5	17.0
5–15 years (%)	30.2	31.7	33.0	34.3	33.9	36.0
Over 15 years (%)	49.2	49.7	49.2	48.9	48.6	47.0
Head of household education level						
Never attended (%)	22.6	21.4	22.8	23.8	21.5	17.4
Some primary (%)	41.2	40.6	38.9	37.5	40.7	43.7
Completed primary (%)	12.0	12.0	14.1	15.3	13.7	14.4
Some secondary or more (%)	24.2	26.1	24.2	23.4	24.1	23.8
Mean household size (SD)	3.9 (1.8)	4.3 (1.8)	4.1 (1.7)	4.0 (1.7)	3.9 (1.7)	4.1 (1.7)
Percent female (%)	54.3	52.7	55.0	54.9	54.9	55.0
Percent of households which own one or more nets^a^ (%)	51.4	83.1	87.5	83.2	81.3	70.9
Percent of SAC who live in households with nets^a^ (%)	55.3	87.5	87.1	84.4	83.2	71.5
Among houses with nets						
Percent of children under five who use nets^a^ (%)	76.2	80.3	87.0	77.6	89.5	68.9
Percent of school-aged children who use nets^a^ (%)	43.6	56.2	68.1	53.9	62.4	46.2
Percent of adults over age 15 who nets^a^ (%)	72.2	72.8	83.1	71.8	82.3	67.0
Ratio of SAC net use to non-SAC net use^b^						
Urban^a^	0.41	0.68	0.55	0.71	0.51	0.50
Rural, moderate transmission	0.47	0.50	0.40	0.45	0.41	0.52
Rural, high transmission	0.51	0.51	0.49	0.36	0.41	0.44

^a^Indicates variable with statistically significant difference (p < 0.0001) between surveys by Chi square test

^b^Prevalence ratio of net use calculated among households that own nets and comparing school-aged children to the remaining population

### Predictors of net use

There were 5781 SAC who lived in households which reported ownership of at least one net and were included in this analysis. Variables significantly associated with increased net use among SAC in bivariate analyses by season were urban or rural—high prevalence transmission settings, younger age, presence of a child under the age of five living in the household, one or more nets per sleeping space in the household, more than half of all nets in the household observed hanging, net source from a government hospital or clinic, average age of nets, increasing head of household education, increasing household wealth index, and decreasing person to net ratio in the household (Table 2).

**Table 2:** Percent of SAC who use a bed net by demographic and household characteristics (N = 7347)

Characteristic	Percent of SAC who use a bed net					
	Rainy ‘12 n = 1048	Dry ‘12 n = 1228	Rainy ‘13 n = 1244	Dry ‘13 n = 1263	Rainy ‘14 n = 1216	Dry ‘14 n = 1348
Sex						
Male	24.0	45.7^a^	57.7	45.8	49.9	30.9
Female	24.3	52.6	60.8	45.3	53.9	34.9
Transmission setting						
Urban	26.5^c^	56.3^c^	59.3^a^	57.3^c^	54.6^c^	44.0^c^
Rural moderate prevalence	15.7	40.4	54.7	33.5	42.3	18.4
Rural high prevalence	29.3	51	63.7	45.9	58.3	36.2
Age						
5–10	28.2^c^	53.4^c^	66.0^c^	48.8^b^	56.7^c^	37.8^c^
11–15	17.2	42.3	48.3	40.3	44.4	25.7
Head of household education level						
Never attended	21.9^a^	44.9^a^	59.6	38.8^c^	48.3	28.4^c^
Some primary	23.8	51.4	57.1	44.3	50.8	28.5
Completed primary	23.5	50.0	59.3	44.4	56.5	37.6
Some secondary or more	35.5	57.6	59.6	59.0	56.5^a^	46.5
Child under age five in the household						
Yes	27.9^c^	48.5	60.4	46.0	53.1	32.4
No	18.2	50.3	57.8	44.9	50.5	33.8
Mean household wealth index						
Lowest	22.8^c^	45.2^c^	59.6	41.1^c^	50.6	26.5^c^
Middle	18.4	47.5	57.9	41.7	52.0	24.8
Highest	36.0	60.9	60.8	56.0	53.2	47.0
Ratio of nets to sleeping spaces						
Less than 1:1	22.8^c^	33.2^c^	39.1^c^	31.53^b^	33.4^c^	22.6^c^
1:1 or more	75.0	71.5	86.4	70.3	83.6	68.0
Ratio of people in house to nets						
More than three people per net	22.5^c^	34.4^c^	37.4^c^	30.6^c^	32.4^c^	27.3^c^
Three people per net	65.6	52.4	77.2	62.1	76.7	53.6
Less than three people per net	77.6	73.5	85.4	71.6	83.3	66.4
Average age of nets in house						
Less than 2 years	40.1^a^	55.7	69.0^a^	55.0	63.9	47.2
Two years or older	49.8	60.6	60.2	50.6	61.2	45.7
Household net characteristics						
Half or less of all nets hanging	33.2^c^	41.2^c^	62.2^b^	45.2^c^	56.0^c^	36.2^c^
More than half of all nets hanging	52.6	72.3	70.9	63.0	66.9	55.1
Majority of nets from government clinic or hospital	44.5^c^	53.1^a^	58.0	49.2	61.0^c^	44.4^c^

^a^Indicates variables which were significantly associated with net use at the 0.05 level in bivariate analysis by season

^b^Indicates variables which were significantly associated with net use at the 0.01 level in bivariate analysis by season

^c^Indicates variables which were significantly associated with net use at the 0.001 level in bivariate analysis by season

*SAC* school-aged children (5–15 years)

Stratification by transmission setting revealed that the association between SAC net use and head of household education level was driven by high net use in urban locations (where a larger portion of the population is highly educated). However, in the 2012 rainy season, a head of household reporting any education (compared to households where the head of household reported no education) was significantly associated with SAC net use in all locations [MH-POR = 1.56 (95 % CI: 1.14, 2.12)]. The association between SAC net use and wealth was similarly a product of high net use in urban locations during the 2012 and 2013 dry seasons. However, being in the highest wealth tertile was significantly associated with SAC net use in rural areas with high malaria prevalence during the rainy seasons of 2012 and 2014; it was also highly associated with net use in all locations during the dry season of 2014 [MH-POR = 2.18 (95 % CI: 1.65, 2.88)]. The presence of a child under the age of five in the household was highly associated with net use by SAC in all locations before universal net distribution. This variable ceased to be significant in any location in the two surveys following universal distribution. However in later surveys, the presence of a child under the age of five was again significantly associated with net use among SAC.

Individual regression models for the predictors of net use among SAC in households that owned at least one net were fit for each survey and, after adjusting for transmission setting, the following were statistically significant at all time-points: age (with children aged 5–10 years significantly more likely to use nets than children aged 11–15 years), ratio of people to nets in a household (SAC living in households with lower ratio of people to nets were more likely to use nets), and higher proportion of nets in a household observed hanging at the time of survey. Additional significant predictors of SAC net use (at some but not all time-points) were female sex, average age of nets in the household, household wealth tertile, and the presence of a child under age five in the household.

In a final model, stratified by transmission setting and adjusting for survey, variables which were significantly correlated with increased net use in SAC in all settings were younger age (5–10 vs 10–15), increasing proportion of nets hanging in a household, decreasing ratio of people to nets, and female sex (Table 3). Additional significant predictors in some but not all transmission settings included the presence of a child under the age of five and increasing household wealth index.

**Table 3:** Final model for correlates of net use among SAC who live in houses with nets (N = 5781)

Covariate	Odds ratios by transmission setting		
	Urban (n = 1948)	Rural—moderate transmission (n = 1725)	Rural—high transmission (n = 2108)
Younger age (5–10)	Ref	Ref	Ref
Older age (10–15)	0.25 (0.19, 0.31)	0.24 (0.18, 0.31)	0.18 (0.14, 0.23)
Half or less of nets in household hanging	Ref	Ref	Ref
More than half of nets in household hanging	8.06 (4.74, 13.70)	3.45 (2.29, 5.22)	5.44 (3.64, 8.15)
Three or more people per net in household	Ref	Ref	Ref
Less than three people per net in a household	4.55 (2.20, 9.42)	4.02 (2.17, 7.44)	3.55 (1.85, 6.81)
No child under age five in household	–	Ref	Ref
Presence of a child less than age five in household	–	1.64 (1.03, 2.61)	2.53 (1.53, 4.18)
Rainy 2012	Ref	Ref	Ref
Dry 2012	3.13 (1.27, 7.72)	0.86 (0.35, 2.13)	0.91 (0.37, 2.20)
Rainy 2013	3.30 (1.34, 8.17)	3.38 (1.37, 8.35)	1.47 (0.60, 3.62)
Dry 2013	2.02 (0.84, 4.87)	1.05 (0.42, 2.58)	0.74 (0.30, 1.80)
Rainy 2014	2.90 (1.19, 7.09)	3.19 (1.26, 8.11)	1.68 (0.68, 4.16)
Dry 2014	1.42 (0.59, 3.44)	0.47 (0.18, 1.21)	0.67 (0.27, 1.67)
Male sex	Ref	Ref	Ref
Female sex	1.44 (1.13, 1.83)	1.34 (1.05, 1.71)	1.55 (1.23, 1.95)
Lowest wealth tertile	2.07 (1.09, 3.94)	2.23 (1.43, 3.49)	–
Middle wealth tertile	Ref	Ref	–
Highest wealth tertile	0.87 (0.50, 1.49)	0.84 (0.48, 1.48)	–

Odds ratios and 95 % confidence intervals from mixed effect logistic regression

All models include survey as a categorical variable; all other variables included only if statistically significant in the model at the p = 0.05 level

*SAC* school-aged children (5–15 years)

### Net use over time

The universal net distribution campaign occurred between the 2012 rainy season (April–May) and dry season (September–October). Household net ownership increased significantly from rainy season 2012 to rainy season 2013 (from 51 to 88 %, p for trend <0.0001) and decreased significantly in each survey after rainy season 2013 (p for trend <0.0001) (Table 1). Overall, net usage among net-owning households increased significantly from the rainy season 2012 survey to the rainy season 2013 survey (63 and 79 %, respectively, p for trend <0.0001) and declined significantly after rainy season 2013 (p for trend <0.0001).

An increase in bed net use among SAC was detected only in the urban setting following the universal bed net campaign (Table 3).

### Age differences in net use over time

**Fig. 1:**
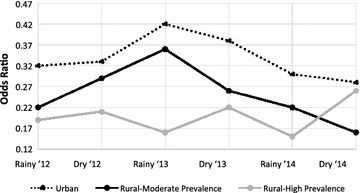
Final model: Odds ratio of net use comparing SAC to all other age groups over time. *OR of net use changes significantly over time (p < 0.001) among participants living in rural areas with moderate transmission. Change over time was non-significant in the urban setting (p = 0.12) and in the rural, high transmission setting (p = 0.14). *SAC* school-aged children (5–15 years), *OR* odds ratio of net use comparing SAC to all others by mixed effect logistic regression

At all time-points SAC had significantly lower net usage than all other ages (p < 0.0001 at all time-points) (Table 1). In the first adjusted model without an interaction term for survey, the OR for net use comparing SAC to all other ages in households that owned at least one net was 0.32 (95 % CI: 0.28, 0.36) in the urban setting, 0.24 (0.21, 0.28) in the rural moderate transmission setting and 0.20 (95 % CI: 0.17, 0.22) in the rural high transmission setting. In the second adjusted model including an interaction term for age and survey, the OR for net use in SAC compared to other age groups only changed significantly over time in the rural areas with moderate transmission (Fig. 1).

## Discussion

Universal ITN distribution in Malawi was initially associated with a population-wide increase in net ownership and use. There was an overall increase in net use among SAC. However, in the areas where net use may be most beneficial (rural, high transmission settings), SAC net use did not significantly increase after the bed net campaign. In urban and moderate transmission rural settings, the increase in net use in SAC after the distribution campaign appeared transient. Other than the distribution campaign, modifiable factors associated with increased use of nets among SAC included an increased ratio of nets to people in a household and a higher proportion of nets in the household observed to be hanging. The presence of a child under the age of five in the household was also associated with net use, likely an indicator that households with SAC gain access to nets as a result of routine distribution to children under the age of five. Non-modifiable factors associated with increased net use among SAC included living in an urban location, being under the age of 11, and female gender.

The data presented herein indicate that, as recommended by the World Health Organization (WHO), frequent universal distribution campaigns are needed to maintain increased population-wide net use. Current distribution strategies in Malawi are limited in their ability to change net use among SAC, the population with the highest prevalence of *Plasmodium falciparum* parasitaemia in the study area. The universal distribution campaign increased bed net use in the entire population and also decreased the disparity in net use between SAC and other age groups in some settings, but this difference was transient. Net use began to decline 1 year after the distribution campaign suggesting that, in Malawi, one-time universal ITN distribution, supplemented with routine distribution through government health centres, may not effectively lead to sustained increases in ITN use. This is in contrast to previous studies that have found that effects of intervention were sustained from 4–6 years [4, 17]; however, no previous studies have looked specifically at the effects of universal ITN distribution on SAC.

In settings where net ownership among the communities surveyed was high (with 88 % of households reporting ownership of one or more nets during the rainy season of 2013), children under the age of five and adults were more than three times as likely to sleep under a bed net than SAC. The factors most strongly associated with net use among SAC were related to the number of bed nets available and in use. When a household had a sufficient number of nets based on the universal distribution goals (at least one net for every two people), SAC were more than twice as likely to have slept under bed nets as SAC in households that fell short of this goal (more than three people per net). Given that SAC are the least likely demographic to use nets, SAC net use may serve as a sensitive indicator of net saturation in a population as opposed to prevalence of household net ownership or net use in children under five.

The influence of a young child in the house on SAC net use suggests that infant-targeted net distribution does have a household-level effect on net use. In households where all the children share a sleeping space, net distribution to children under five may also increase access to nets among older children. However, if the majority of nets are entering the household through under-five clinics and older children are sleeping separately, novel distribution strategies are needed to effectively reach SAC. With current net distribution strategies, households without younger children or pregnant women have no consistent source of free ITNs. Innovative methods of net distribution targeting SAC, such as school-based campaigns, are necessary to address the net use disparity.

The unique qualities of this study design may limit the ability to capture the true effect of universal net distribution. Despite having multiple time-points, this data is cross-sectional and although survey staff returned to the same location for each survey, they did not collect individual identifiers and analysis was unable to account for the possibility of surveying the same individuals over time. Additionally, these data were collected as part of a surveillance programme without control arms and with only one season of pre-intervention data. Despite these limitations, this study was able to identify clear trends in predictors of net use over the 3 years surveyed. The clear pattern of changes seen following the bed net campaign and the consistency of these results across transmission settings, support the validity of the findings.

## Conclusions

Net use among children aged 5–15 is dependent on the ratio of nets to people in a household and SAC net use may serve as a better indicator of sufficient net distribution than prevalence of household net ownership. While mass distribution campaigns have a temporary effect on net use in the overall population, this study supports the WHO recommendations that universal net distribution campaigns be repeated at frequent intervals. Additional innovative distribution strategies targeting SAC are needed to address the deficiencies in net use among this population.

## Abbreviations

CI: confidence interval
EA: enumeration area
IRS: indoor residual spraying
ITN: insecticide-treated net
IRS: indoor residual spraying
LLIN: long lasting insecticidal net
MH-POR: Mantel–Haenszel prevalence odds ratio
MIS: malaria indicator survey
OR: odds ratio
SAC: school-aged children
WHO: World Health Organization
